# Pre-training via Transfer Learning and Pretext Learning a Convolutional Neural Network for Automated Assessments of Clinical PET Image Quality

**DOI:** 10.1109/TRPMS.2022.3231702

**Published:** 2023-04

**Authors:** Jessica B. Hopson, Radhouene Neji, Joel T. Dunn, Colm J McGinnity, Anthime Flaus, Andrew J. Reader, Alexander Hammers

**Affiliations:** Department of Biomedical Engineering, King’s College London; Siemens Healthcare Limited; King’s College London & Guy’s and St Thomas’ PET Centre, King’s College London; King’s College London & Guy’s and St Thomas’ PET Centre, King’s College London; King’s College London & Guy’s and St Thomas’ PET Centre, King’s College London; Department of Biomedical Engineering, King’s College London; King’s College London & Guy’s and St Thomas’ PET Centre, King’s College London

**Keywords:** Convolutional neural networks, Deep learning, Image quality, Image reconstruction, Transfer learning

## Abstract

Positron emission tomography (PET) using a fraction of the usual injected dose would reduce the amount of radioligand needed, as well as the radiation dose to patients and staff, but would compromise reconstructed image quality. For performing the same clinical tasks with such images, a clinical (rather than numerical) image quality assessment is essential. This process can be automated with convolutional neural networks (CNNs). However, the scarcity of clinical quality readings is a challenge. We hypothesise that exploiting easily available quantitative information in pretext learning tasks or using established pre-trained networks could improve CNN performance for predicting clinical assessments with limited data. CNNs were pre-trained to predict injected dose from image patches extracted from eight real patient datasets, reconstructed using between 0.5%-100% of the available data. Transfer learning with seven different patients was used to predict three clinically-scored quality metrics ranging from 0-3: global quality rating, pattern recognition and diagnostic confidence. This was compared to pre-training via a VGG16 network at varying pre-training levels. Pre-training improved test performance for this task: the mean absolute error of 0.53 (compared to 0.87 without pre-training), was within clinical scoring uncertainty. Future work may include using the CNN for novel reconstruction methods performance assessment.

## I Introduction

POSITRON emission tomography (PET) is an important modality in the management of several brain diseases, notably memory problems, dementia, epilepsy [1], and brain tumours [2]. Simultaneous positron emission tomography − magnetic resonance imaging (PET-MR) has potential to become an important tool in the diagnosis of memory clinic patients [3], by simultaneously providing both functional and structural information of the brain [4]. However, a drawback to PET imaging is the need for administration of the radioligand: the more radioligand injected, the higher the associated radiation dose, and radioligand production to Good Manufacturing Practice standards is very expensive [5]. Both can be targeted by reducing the injected dose [6], but at the expense of the reconstructed image quality, caused by increased noise and a loss of resolution [7]. Traditionally, image quality assessment has relied on human raters, but this is not scalable and impractical for large-scale investigations or repeating assessments while optimising reconstruction algorithms. Thus, it is beneficial to determine automatically whether a reconstructed image is of satisfactory diagnostic quality. One approach is to use convolutional neural networks (CNNs), as they are data-driven [8] and versatile, being used for medical imaging tasks including image segmentation, lesion detection and image de-noising [9]–[14]. CNNs have also shown to outperform radiomic analysis in certain tasks [15].

A drawback of using CNNs is that generally a large dataset is required for training [16]. However, this is challenging in medical imaging, as clinically-annotated data are scarce and not scalable [17]. Associated acquisition and processing costs contribute to the paucity of clinician-labelled data. Pre-training (an architecture trained on a separate image database) and transfer learning (taking features learned on one problem and using them for a new problem) could be exploited to overcome this lack of training data [18] [19]. Transfer learning can be approached with either fine tuning or fixed representations [20]. Fine tuning involves unfreezing some or all of the network parameters in order to update the weights with a very low learning rate and a new dataset [18].

Pre-training with natural image databases has been used in the medical imaging field. ImageNet [21] is a dataset of more than 15 million high-resolution natural images annotated for 22,000 classes. A subset of ImageNet used for deep learning tasks consists of 1.2 million images categorised into 1000 classes [22]. Pre-trained networks via ImageNet have been used for the binary classification of papillary thyroid carcinoma [23], where exploiting a VGG16 [24] network achieved a 97.66% accuracy when distinguishing between carcinomas and benign thyroid nodules in two-dimensional cytological image patches. Dunnmon *et al*. [25] showed that CNNs could be pre-trained on ImageNet to classify chest radiographs into “normal” or “abnormal” with an area under the receiver operating characteristic curve (AUC) of 0.96. This was built upon by Tang *et al*. [26], who investigated a greater number of established architectures, including VGGNets [24] and Inception-v3 networks [27], and compared pre-trained networks against training from scratch. Their work showed that when using a moderately sized dataset (8500 two-dimensional chest radiographs), pre-training on ImageNet out-performed training the model from scratch. It has also been shown that a transfer-learned VGG16 network pre-trained via ImageNet outperformed an Inception-v3 network for the task of classifying into ‘Normal’ or ‘Pneumonia present’, and matching the complexity of the model to the dataset size along with data augmentation, further improved CNN accuracy [28].

However, there is debate on the usefulness of transfer learning from a natural image domain to a medical imaging domain [29], [30]. Recent work on transfer learning questions the validity of using natural images for pre-training in medical imaging tasks due to the high dissimilarity between the domains [31]. Heker *et al*. [32] also showed transfer learning within domain (from one medical image dataset to another) outperformed inter-domain pre-training (from a natural image database to a medical image database) for segmentation and classification tasks.

Pre-training can be used to overcome extensive training data needs. Mustafa *et al*. [19] showed network performance could be improved by increasing size of both the model architecture, and the natural image pre-training dataset. ImageNet-21k (the full ImageNet dataset classified into 21000 classes) obtained superior performance over ImageNet (the subset of ImageNet classified into 1000 classes). However, the full dataset is more computationally expensive and labels are not mutually exclusive: a single image may have multiple labels, making classification more difficult [33].

As pre-trained networks act as feature extractors, layers need to be frozen to prevent the network from retraining too extensively, potentially negating any pre-training benefit. As shown in [34], inclusion of multiple layers in the fine tuning process can improve model performance. In an Alzheimer’s disease diagnosis classification task, all convolutional layers of pre-trained networks were frozen, except for the last fully connected layer which was trained on the new dataset [35]. Kieffer *et al*. [36] retrained the last convolutional block and fully connected layer of a VGG16 network for their dissimilar domains.

Whilst there have been many image quality assessment investigations in the computer vision field [37]–[39], similar experiments within the medical imaging field have emphasised binary classifications. One such investigation by Sujit *et al*. [40] used a CNN to predict image quality of brain MRIs compared to visual evaluation by two experts by scoring the images as ‘0 (acceptable)’ or ‘1 (unacceptable)’, achieving an accuracy of 84%. Oksuz *et al*. [41] used cardiac MR images to detect motion-related artefacts, classifying the image as “good” or “poor”. Similarly, Ma *et al*. [42] used a CNN to carry out both a binary classification (‘0 − non-diagnostic’ or ‘1 − diagnostic’) and three-class classification (‘0 − poor/non-diagnostic’, ‘1 − diagnostic’ or ‘2 − excellent’) of MR images, achieving an accuracy of 84% and 65%, respectively. In the field of retinopathy, a similar approach was taken by Coyner *et al*. [43] to determine whether clinically-scored 2D images were of “acceptable”, “possibly acceptable” or “not acceptable” quality, and they found a strong correlation (Spearman’s rank correlation coefficient of 0.84 − 0.92) between the CNN and the expert scoring. Another retinopathy study showed that pre-training via ImageNet outperformed training from scratch [44].

Building on previous work [45], the aim of this work was to assess the influence of transfer learning as a function of the number of fixed parameters during training, as well as comparing different pre-trained architectures for the prediction of clinical PET image quality assessments. Most of the literature relates to CT and MR images, and there is limited research on PET image quality assessment. Varying image qualities were generated by reconstructing images with different simulated injected doses such that all other factors that may affect image quality, including reconstruction algorithm and system design, were fixed, and only the dose level determined the differences in image quality. The performance of an established VGG16 network [24] pre-trained on the ImageNet-1K database [21] was assessed and compared to a pre-text learning task (using an easily extracted label, the injected dose) as a form of pre-training to predict three clinically scored metrics. These metrics are: global quality rating (GQR), which evaluates the aesthetic component of the reconstruction, and takes into consideration qualities such as noise level and resolution; pattern recognition (PR), which determines whether the reconstruction allows for the clinician to determine the presence of any pathological patterns suggesting a particular diagnosis; and diagnostic confidence (DC), which concerns the certainty with which the clinician can use the reconstruction to observe these patterns and make a diagnosis. As low count reconstruction causes the degradation of PET image quality, these metrics were designed by the authors to encompass the necessary considerations involved in using these low count clinical PET images for making a confident and accurate diagnosis. These three metrics are scored as: 0 = “unacceptable”, 1 = “poor but usable”, 2 = “acceptable” and 3 = “good/excellent”, with 0.5 ratings accepted. The aim was to reduce the need for clinically-assessed training data by the use of pre-trained networks and transfer learning.

## II Methods and materials

### A Real Patient Data

**Fig. 1:**
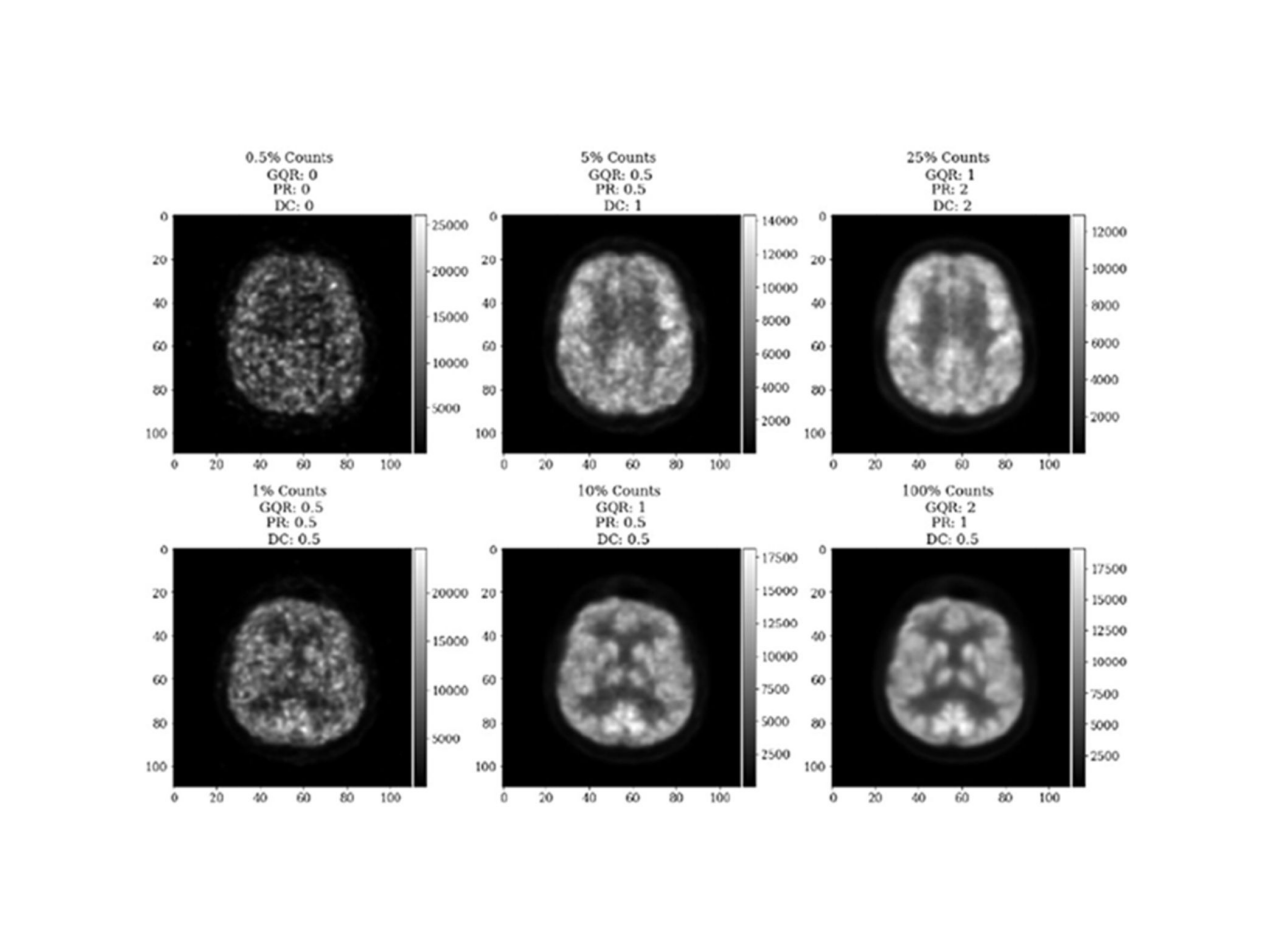
Cropped example transverse slices from “low”, “medium” and “high” quality reconstructions for two independent datasets.

**Fig. 2:**
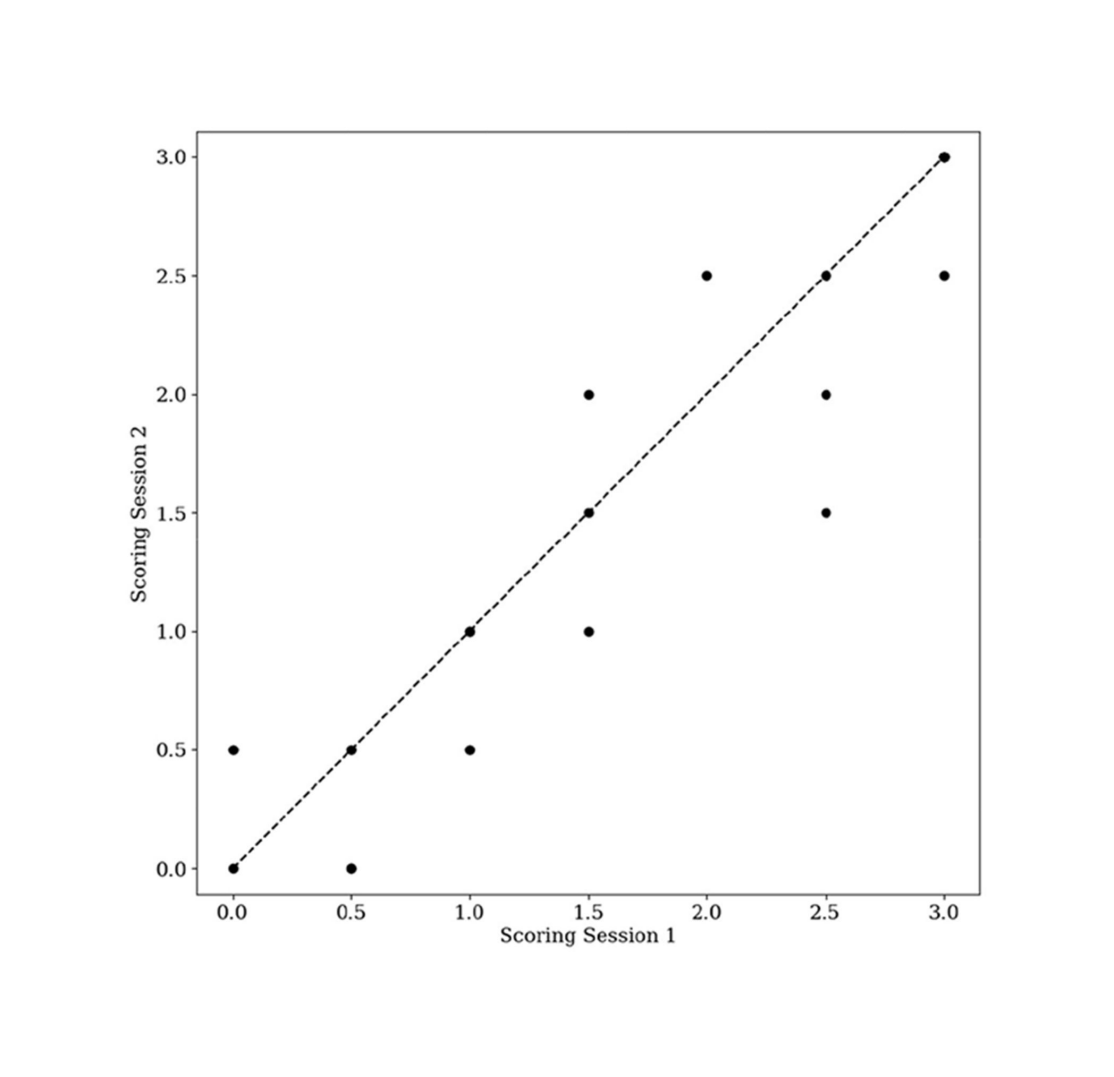
Inter-session scoring difference for all clinical metrics.The dotted line is the identity line for scoring session 1.

**Fig. 3:**
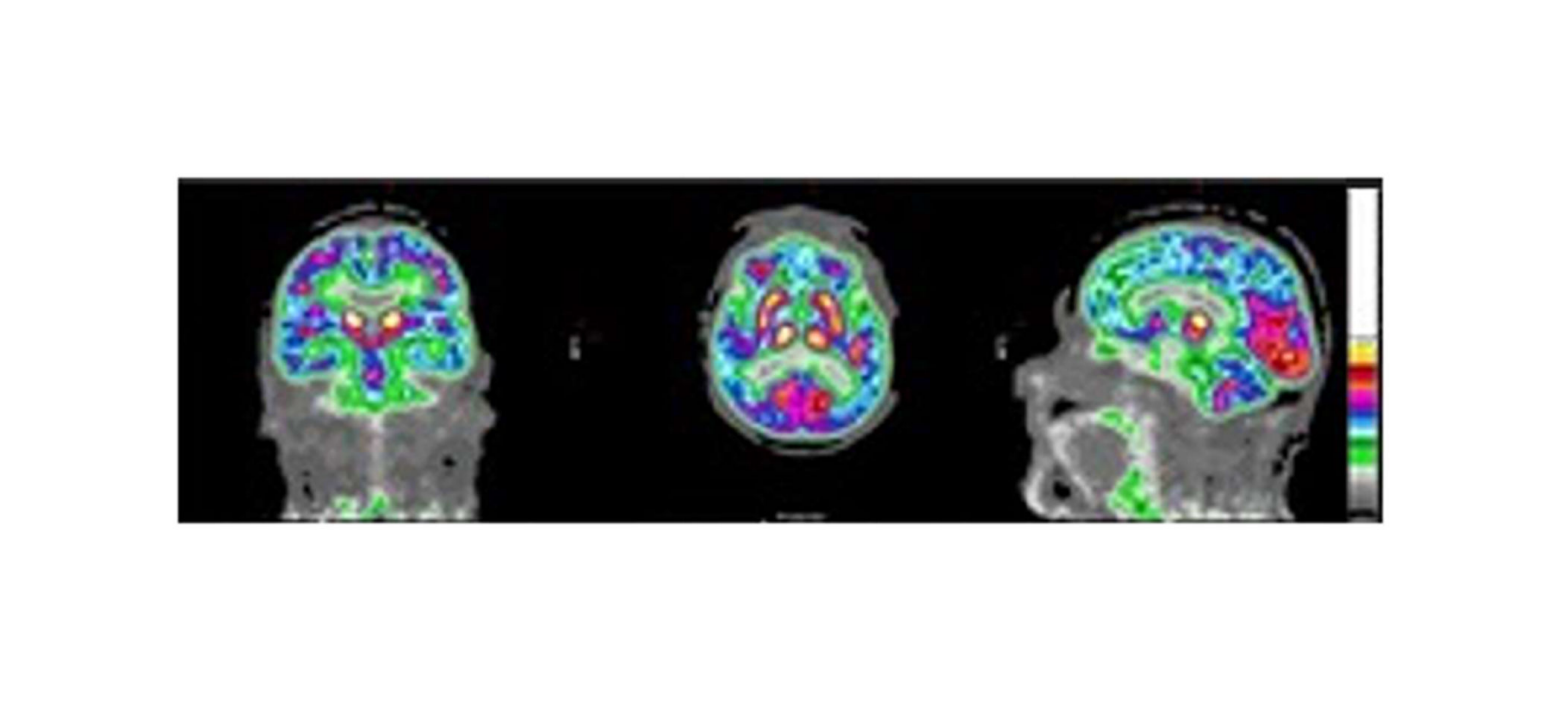
A clinically assessed image with a 20-point colour scale with a lower global quality rating (1), but higher pattern recognition (2) and diagnostic confidence (2) scores (corresponding to the top-right subplot in Fig. 1). The colour-scale is the technique by which the clinician determines the pattern recognition and diagnostic confidence ratings.

Data from PET-MR scans of 21 memory clinic patients with suspected dementia were used. Each patient underwent an [^18^F]FDG-PET and a simultaneous 3D T1-magnetisation-prepared gradient-echo (MP-RAGE) MRI scan on a 3T PET-MR scanner (Biograph mMR, Siemens Healthcare, Erlangen, Germany). Siemens e7 tools was used for image reconstruction, using the clinical standard of the ordered subset expectation maximisation (OSEM) algorithm with 2 iterations and 21 subsets to reconstruct 3D images of 344 × 344 × 127 voxels. It is possible for the list-mode PET data to be resampled [45], [46], and such methods can be used to simulate seven lower injected doses (Fig. 1). To simulate the lower injected doses, the prompts and random coincidences in the corresponding list-mode PET data for the 100% count reconstruction were randomly sampled. Subsequently, the corresponding emission, normalisation, randoms and sinograms were re-calculated along with the attenuation sinograms using Siemens e7 tools. The list-mode PET data from all datasets were resampled [46], simulating seven lower injected doses (Fig. 1). Images were normalised between 0-1 to reduce standard deviations, suppressing the impact of outliers, then blinded and randomised prior to clinician scoring. The simulated dose levels were grouped into “low quality” (0.5% and 1% of counts), “medium quality” (5% and 10% of counts), and “high quality” (25%, 50% and 100% of counts) categories to decrease clinical assessment times. In total, ten patients at three different count levels (one per category) were assessed by an experienced clinician. For calibration, nine images were scored twice by the same clinician in different scoring sessions (Fig. 2). Another experienced clinician also scored the same images. Pearson’s correlation coefficients of 0.91, 0.91, 0.90 for GQR, PR and DC, respectively, were found between the clinicians’ scores. It was also found that 91% of the scores were within 0.5 of that given by clinician 1, which is within the calibration range (Fig. 2) showing the robustness of the scores given by each of the clinicians. After consensus readings, only one score was greater than 0.5 from clinician one’s original scoring, thus clinician one’s scores were used for this study. Fig. 3 shows the 20-point discrete colour scale used during the scoring process which was optimised for reading brain PET images at the Guy’s and St Thomas’ PET Centre. This colour scale allows for the specific pathological patterns to be more easily highlighted, such that with a lower quality image, a diagnosis could still be made, helping to determine pattern recognition and diagnostic confidence. The image used in Fig. 3 corresponds to the same example in the top right subplot in Fig. 1. Table I shows a Pearson’s pairwise correlation coefficient for each of the metric scores given by both clinicians.

### B Extraction and Thresholding of Patches

For each patient, 1000 patches of size 80 × 80 pixels were extracted at random from transverse, sagittal and coronal planes to cover the whole 3D volume, whilst not being too computationally exhaustive. Patches instead of whole images were also used as a form of data augmentation, as has been shown to improve model performance [28]. To eliminate background-only patches, a thresholding algorithm was applied to each individual patch. If the average pixel value of the patch was below one-eighth of the average pixel value of the whole image volume, then the patch was rejected as background, ensuring that only patches with meaningful brain information were used, as is used when defining brain masks in FDG PET imaging [47]. Of these thresholded patches, 100 each from the transverse, sagittal and coronal planes, were randomly sampled, such that 300 patches were used for each reconstructed image volume. This number of patches was chosen as it is proportional to the brain:background ratio in the whole image volume.

### C Model Architecture

**Fig. 4:**
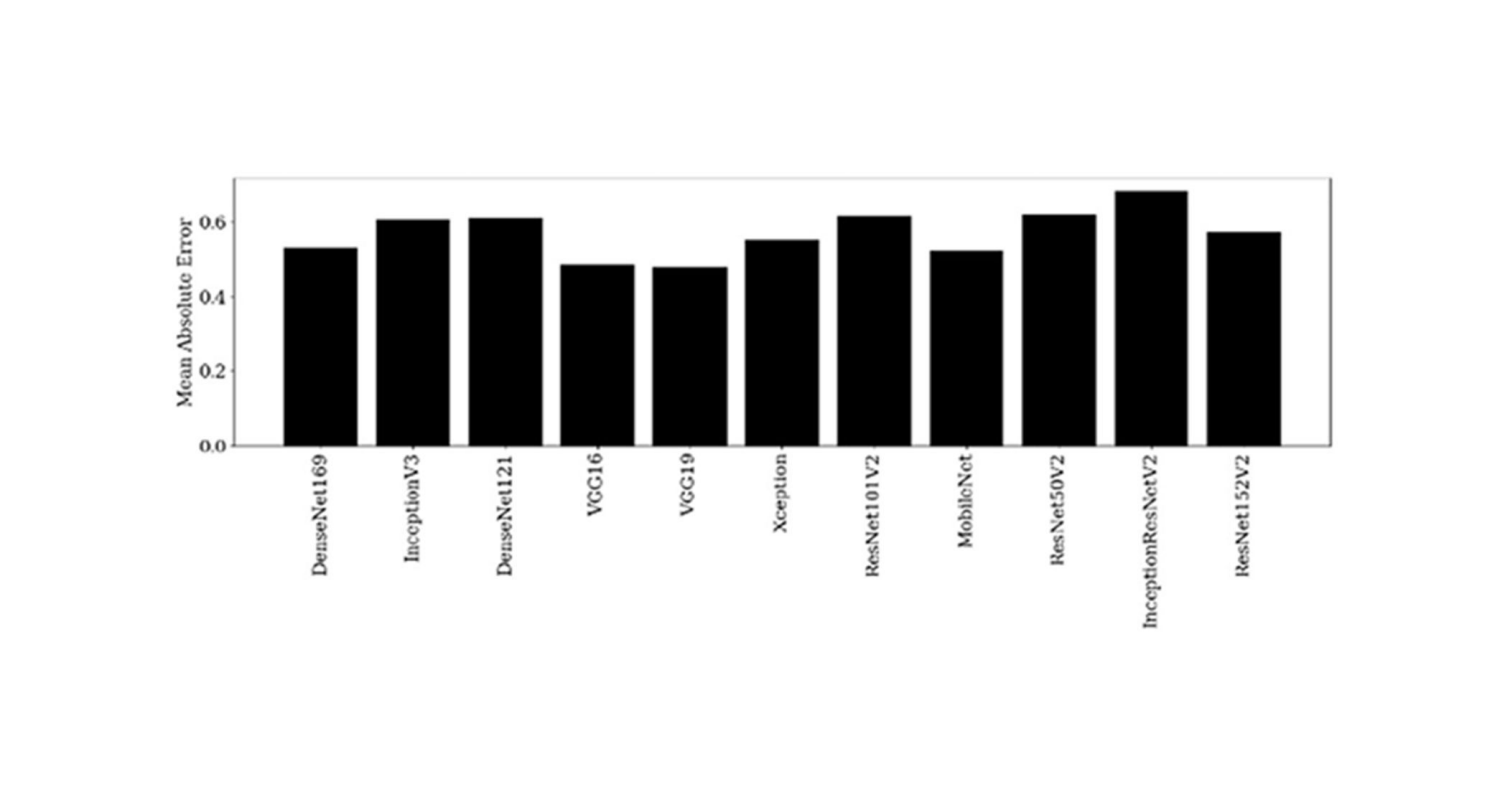
Bar chart of the mean absolute error based on the same three test patients for 11 established CNN backbones for three training runs. A VGG16 backbone is shown to be representative of other established CNN backbones

**Fig. 5:**
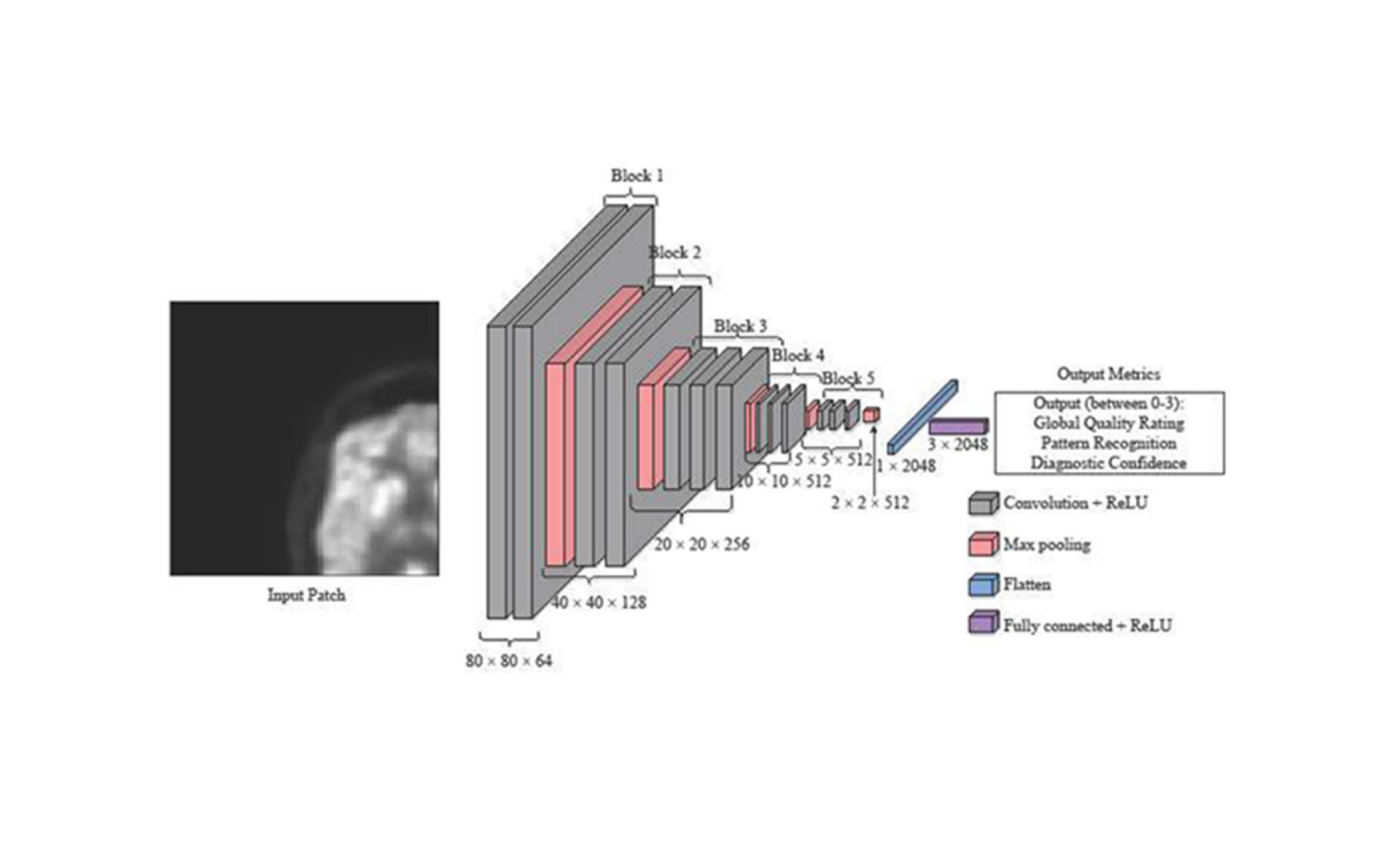
Transfer learned VGG16 architecture. A patch of size 80 × 80 is used as the input with the three quality metrics as output. Each of the five convolutional blocks is labelled.

A VGG16 architecture [23] with random weight initialisation (Section II.D) or ImageNet [20] weight initialisation (Sections II.D and II.E) acted as a feature extractor. In addition to the VGG16 backbone being used for other medical imaging tasks [28], [36] and its block structure providing a demonstrable method of pre-training levels, the VGG16 backbone was chosen as it is representative of similar CNN architectures (Fig. 4). A total of 26 different established backbones were trained on the same dataset, with the minimum validation loss monitored. The eleven models with the lowest validation losses, were trained another two times, and were then tested on the same three test patients (Fig. 4). There are five convolutional blocks consisting of convolution layers and a max pooling layer (Fig. 5). The three original fully connected layers of the VGG16 architecture were replaced with a single fully connected layer with three outputs relating to each of the clinical metrics (GQR, PR and DC) with ReLU activation (Fig. 5) in Section II.D. This is because the original VGG16 network is used for classification of the ImageNet database into 1000 different classes. However, this is a regression task, with the output a prediction of three clinical quality scores. Therefore, the original projection head was replaced by another fully connected layer to achieve this task. In Section II.E, the replacement fully connected layer had a single output corresponding to the simulated dose. The same seven patients were used in the transfer learning dataset, reconstructed at three simulated dose levels each. Greyscale input patches were replicated along one axis to obtain three channels as VGG16 inputs are RGB images.

### D Transfer Learning: Pre-training a VGG16 Backbone

The VGG16 network (Fig.5) was initialised with random weights, with a learning rate of either 10^-3^ (default) or 10^-4^ as a baseline comparison model.

The VGG16 network was then also initialised using ImageNet weights, and trained by sequentially unfreezing weights from consecutive blocks starting from training zero convolutional blocks (only the fully connected layer) up to and including all five convolutional blocks. Each model was trained seven times and seven patients were used in the transfer learning dataset. This model was also tested as a function of the number of patients in the transfer learning dataset; a maximum of 6300 patches were used for transfer learning.

### E Pretext Learning: Injected Dose Inference

**Fig. 6:**
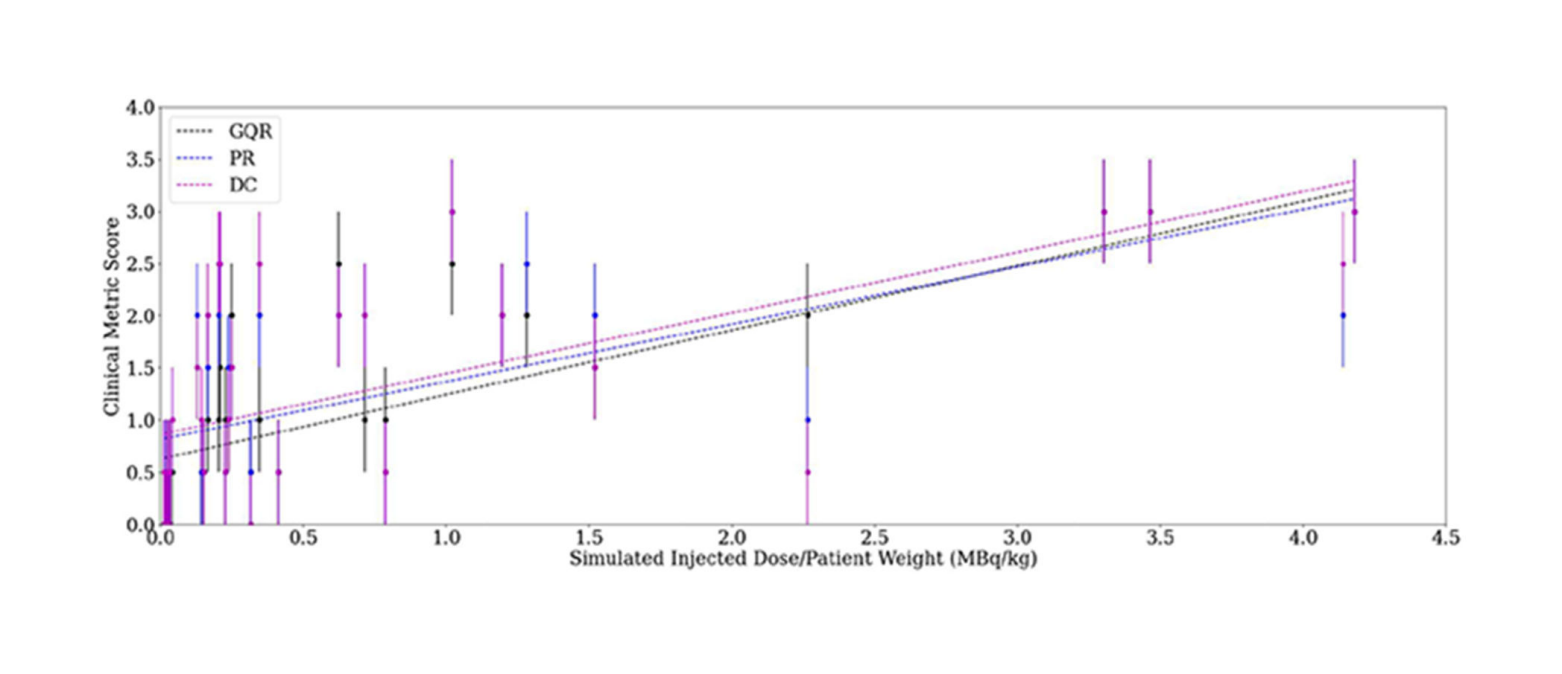
Correlation between clinically-scored metrics and injected dose standardised by patient weight. Dotted line = line of best fit. Error bars = ± 0.5, informed by Fig. 2

**Fig. 7:**
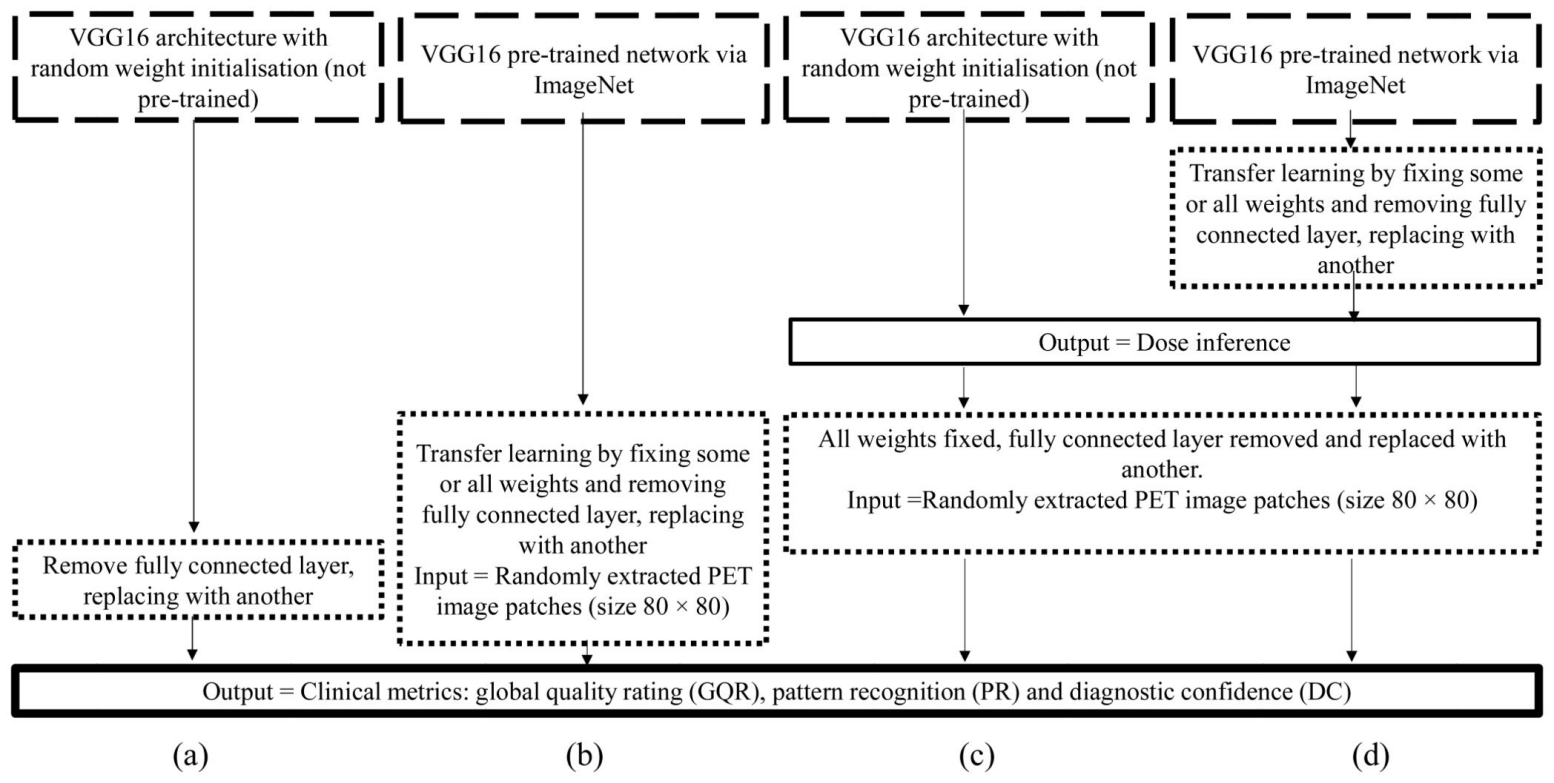
Pipelines of pre-training. (a) VGG16 architecture without pre-training (random weight initialisation). (b) Transfer-learned VGG16 backbone pre-trained on ImageNet to predict the same three clinical metrics. (c) VGG16 backbone with random weight initialisation to initially infer dose as a pretext learning task, with weights from this model fixed and the fully connected layer removed and replaced with another to output the prediction of the three clinical quality metrics. (d) VGG16 backbone with ImageNet weights to initially infer dose as a pretext learning task, with weights from this model fixed and the fully connected layer removed and replaced with another to output the prediction of the three clinical quality metrics.

Dose inference was used as a pre-text learning task due to the positive correlation between the number of counts in the reconstruction and the clinician-assigned score (Fig. 6). Patient weight was used to standardise between patients. In total, eight patients (16,800 patches) were used for training this network, with another patient used for validation. Both pre-trained (via ImageNet and re-training of the last two convolutional blocks) and not pre-trained networks (via random weight initialisation and re-training either the last two or all five convolutional blocks) were compared. For additional comparison, the dose standardised by patient weight was used as input into a model consisting of a fully connected layer with the three clinical metrics as the output. All pipelines are shown in Fig. 7.

### F Evaluation Metrics

Three evaluation metrics were used: mean absolute error (MAE) between the predicted and true clinical scores; percentage exact agreement (predicted score = true score); and percentage close agreement (predicted score = true score ± 0.5), in line with Fig. 2. All models were tested on the same three test patients at three dose levels each.

### G Implementation

Reconstructions via Siemens e7 tools were carried out in MATLAB (The Mathworks, Inc.). All models were trained using the Keras [18] application programming interface implemented in TensorFlow [48]. Training was accelerated using either an NVIDIA Tesla K40 12GB GPU or NVIDIA Quadro RTX6000 24GB GPU. All models used the Adam optimiser with a learning rate of 10^-4^ (unless otherwise stated), a mean-squared-error loss function and batch size of 10. Training occurred for 1000 epochs, and models were saved at the lowest validation loss which was used for analysis.

## III Results

### A Influence of the Number of Retrained Blocks

**Fig. 8:**
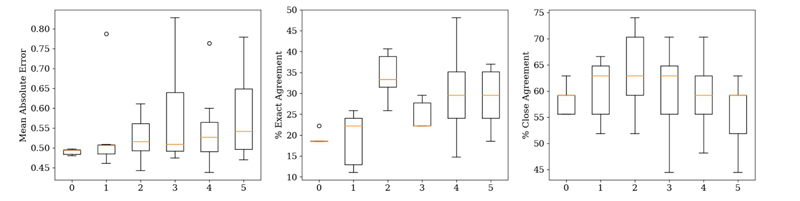
Boxplots of MAE, percentage exact agreement and percentage close agreement as a function of the number of pre-fully connected layer convolutional blocks of the VGG16 backbone retrained (0 to 5). Left: 0 - only the fully connected layer trained; Right: 5 - all weights updated. Plotted across all clinical metrics for seven training runs. The middle line is the median.

Based on the three test patients, Fig. 8 shows a series of boxplots describing the evaluation metrics as a function of the number of pre-fully connected layer blocks when seven patients at three dose levels each were used in the transfer learning dataset. Re-training the last one or two blocks outperformed training the fully connected layer only, but there was no clear benefit to retraining the last 3-5 blocks, as percentage close agreement decreased. Re-training a higher number of convolutional blocks, generally increased the variation across separate training runs, without further decreases in the mean or median absolute error. There is a plateau in the MAE around 0.5, in line with Fig. 2.

### B Influence of the Number of Patients in the Transfer Learning Dataset

**Fig. 9:**
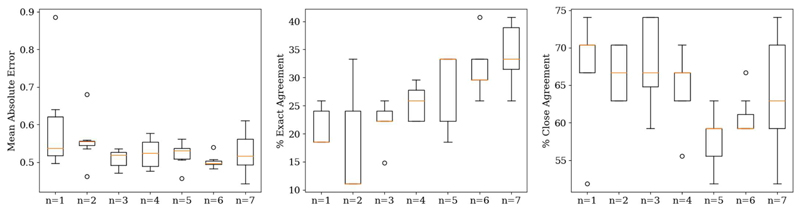
Boxplots of the mean absolute error, percentage exact agreement and percentage close agreement as a function of the number of separate patients in the transfer learning datasets (*n=1* to *n=7*). Plotted across all clinical metrics for seven training runs. The middle line is the median. The model used in for this experiment is that in Fig. 7b with the last 2 convolutional blocks trained.

Fig. 9 shows a series of boxplots describing the same metrics as in Fig. 8. Based on Section III.A, the last two convolutional blocks of the VGG16 backbone were re-trained. Generally, the MAE and the associated variation decreased with increasing numbers of independent training datasets, but plateaued around 0.5, again in line with Fig. 2. Using one patient in the transfer learning dataset was consistently outperformed across all evaluation metrics by more independent patients in the transfer learning. There was clear improvement in the percentage exact agreement with increasing numbers of independent training patients.

### C Dose Inference Performance as a Function of Pre-Training and Retraining

Table II shows the MAE and percentage exact agreement comparing the different models investigated for analysing the pretext dose inference task (before transfer learning to clinical metric prediction), assessing the validation patient reconstructed at seven different dose levels. The green boxes indicate the best value across the three clinical metrics. Pre-training VGG16 with ImageNet and retraining the last two blocks vastly outperformed the VGG16 network with random weight initialisation and retraining all five convolutional blocks. Table III also shows the impact of using only the easily accessible dose label as input into a single fully connected layer, for a learning rate of both 10^-3^ and 10^-4^, with the output as the prediction of the three clinical metrics. The green boxes indicate the best value across the three clinical metrics; lighter green boxes indicate a draw.

### D Comparison of all Pipelines

**Fig. 10:**
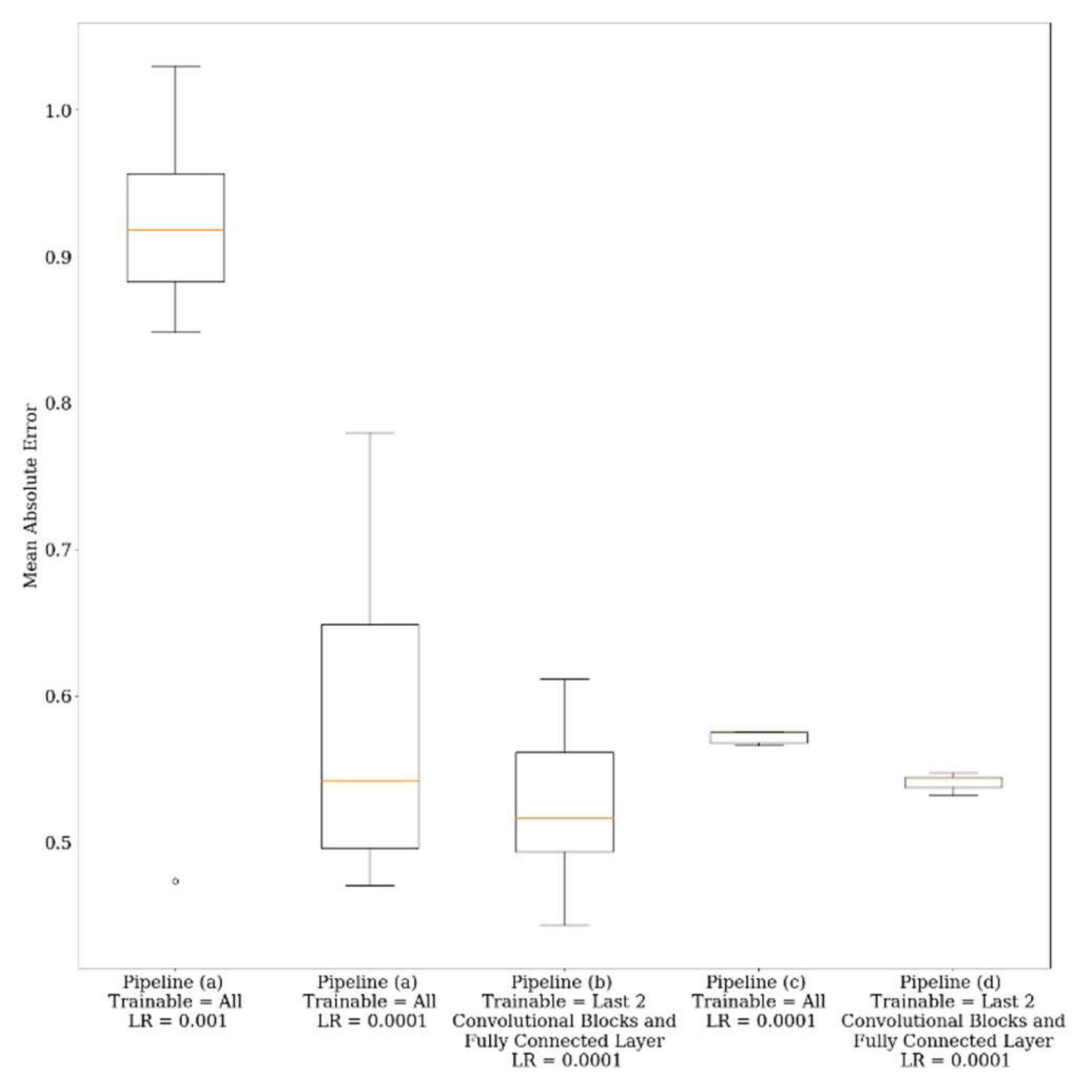
Comparison boxplots of the MAE across all clinical metrics for each methodology over seven separate training runs. LR = Learning Rate. Pipeline (a) corresponds to training the VGG16 backbone with random weight initialisation; pipeline (b) involves training the VGG16 backbone with ImageNet weight initialisation, in this case, training last two convolutional blocks as the fully connected layer; pipeline (c) corresponds to using a pretext learning task of dose inference with random weight initialisation; and pipeline (d) is the dose inference pretext learning task with ImageNet weight initialisation.

Fig. 10 compares all pipelines; from left to right, the amount of pre-training increases, starting from no pre-training (Fig. 7a), to pre-training via ImageNet (Fig. 7b), to transfer learning from the pretext learning task with random weight initialisation (Fig. 7c), and subsequently with ImageNet weight initialisation (Fig. 7d). No pre-training with a learning rate of 10^-3^ was outperformed by all other models. Whilst reducing the learning rate improved MAE 0.87 to 0.58, including ImageNet weights and freezing the first three convolutional blocks further reduced MAE to 0.53. Adding another layer of pre-training via the pretext learning task with random weight initialisation, reduced variation over the seven separate training runs. Initialising this model with ImageNet weights, slightly reduced the MAE compared to when random weight initialisation was used; however, MAE between all pre-trained models were comparable. Table IV compares the same models as in Fig. 10, but evaluated across individual clinical metrics for each evaluation metric and the Spearman’s rank correlation coefficient. Bright green boxes indicate the best value for each evaluation metric, whilst lighter green boxes indicate the second best performing network. The majority of the best values for each metric were achieved by a pre-trained model. Generally, GQR is predicted more accurately than PR and DC.

## IV Discussion

There is a strong correlation between the metrics (Table I), especially between PR and DC, but these metrics were designed to specifically incorporate what the clinician is observing in order to determine if a decreasing image quality may still lead to an image that is clinically useful for making a diagnosis. The VGG16 backbone was chosen as it representative of other established CNNs (Fig. 4). Testing the VGG16 backbone as a function of the number of trainable convolutional blocks shows the necessary level of pre-training required for a better performance on unseen patients. The expected trend in Fig. 8 is that of a “U-shape” for MAE and an “inverted U-shape” for the percentage agreement metrics. At either extreme (either 0 or all 5 of the convolutional blocks trained), there should be a decreased performance (higher MAE and lower percentage exact and close agreements). This is because when none of the convolutional blocks are trained, there remain high-level features specific to the ImageNet dataset and not to medical images, and similarly, when all parameters are trainable, this negates any pre-training benefit [49]. Unfreezing the final two convolutional blocks’ weights resulted in the best percentage exact and close agreements, suggesting a balance between the number of high level features and pre-training benefit, agreeing with the work of Yosinski *et al*. [49].

Fig. 8 informed the investigation into the number of patients in the transfer learning dataset where the last two convolutional blocks of the VGG16 backbone were re-trained. The expected trend was that with more patients in the transfer learning dataset, the better the network performance on unseen datasets as there are more examples from which the model can learn [50]. Fig. 9 indeed shows that increasing the number of patients in the training dataset improved the percentage exact agreement and decreased the MAE, supporting the idea that a larger dataset can improve generalisability to unseen datasets. Dunnmon *et al*. [25] showed that increasing the number of 2D chest X-ray images from 2000 to 20,000 improved the average area under the receiver operating characteristic curve (AUC) from 0.84 to 0.95, but improvements plateaued afterwards, as using 200,000 images, only achieved a non-significant increase in AUC to 0.96. Results from the use of just one patient in the transfer learning dataset were consistently outperformed by using ≥2 patients, suggesting that use of one patient in the dataset was insufficient to generalise to unseen data. The percentage close agreement started to plateau at ~70% beyond ≥3 independent training patients, but the percentage exact agreement continually improved. This suggests that, whilst three patients are sufficient for predictions within 0.5 of the clinician-scores, a larger dataset (i.e. >7) may be required to achieve exact agreements between the true and predicted scores.

For both Fig. 8 and Fig. 9, the MAE plateaued at ~0.5 corresponding to the inter-session scoring uncertainty within the clinical assessments of the images (Fig. 2). A total of 27 images were re-assessed by the same clinician, with 44% of the readings scored at least 0.5 different to the original assessments. Thus, obtaining a MAE of approximately 0.5 may be the optimum achievable for this task.

Table II shows that using pre-training via ImageNet outperformed no pre-training for dose inference. Informed by Fig. 8, pre-training VGG16 with ImageNet weights and retraining the last two blocks was used as the pre-trained network. The pre-trained network vastly outperformed VGG16 with random weight initialisation and training all five convolutional blocks, confirming the benefit of using pre-training instead of training from scratch for this task. The MAE was substantially lower than in the clinical score prediction task, possibly because 56 individual images were available compared to 21 clinically-assessed images.

Comparing all pipelines, Fig. 10 and Table IV show that pre-training provided a benefit to the overall network performance on unseen test data. Fig. 10 shows the MAE for no pre-training was ~0.92 when the default learning rate of 10^-3^ was used, suggesting that the network did not generalise well to unseen data. By simply decreasing the learning rate to 10^-4^, the MAE was substantially reduced to 0.58. Subsequently changing this model to include ImageNet weight initialisation and only re-training the last two convolutional blocks together with the new fully connected layer resulted in decreases in the MAE across all predicted clinical metrics and test patients, i.e. pre-training via ImageNet provided an improvement to overall model performance at test-time. Introducing the dose inference pretext learning task with no pre-training (all parameters trainable and random weight initialisation), reduced the variation between runs, suggesting the dose inference pretext learning task was the source of uncertainty reduction. Using pre-training via ImageNet weight initialisation, again reduced the MAE compared to the same model with random weight initialisation, indicating that by adding another level of pre-training, model performance could be further improved (if minimally). Table IV shows that using a pretext learning task had an inferior performance than transfer learning straight from ImageNet. This is perhaps due to dose inference being more difficult when the images are normalised, as the dose information contained in the overall activity levels is eliminated, and ImageNet provides more complexity as a feature extractor, whereas when using the dose inference network, there is only one output from which the transfer-learned model is trying to learn. Whilst there was a comparable MAE across all three clinical metrics (Fig. 10) for all pipelines, it is arguable that transfer learning directly from a VGG16 backbone, as opposed to carrying out a pretext learning task, is more favourable, as additional patient datasets are not required.

The VGG16 backbone with random initialisation achieved the best performance for two of the 12 evaluation metrics (Table IV), but only narrowly so. However, when ImageNet weights were added, this resulted in best performance for 7 out of 12 evaluation metrics, often by a large margin.

Reducing the learning rate to 10^-4^ from 10^-3^ improved model performance across all evaluation metrics. This suggests that at the higher learning rate, training loss converges too early, such that when early stopping is employed, the model did not generalise well to unseen data. Using no pre-training with a reduced learning rate performed best for 2 out of 12 evaluation metrics (Table IV), namely for PR and DC. This may indicate that the VGG16 architecture was sufficient as a feature extractor for these metrics. However, the metrics’ standard deviations overlapped between this model and using ImageNet for pre-training, and GQR prediction was inferior. The Spearman’s rank correlation coefficients were also consistently highest for pre-trained networks, suggesting an overall better performance for predicting clinician scores.

Table IV supports Fig. 10 and shows that generally, GQR is more closely predicted to the true score than PR and DC. This may be because the global quality of an image is much easier to predict, as the model can estimate noise level. It is well-known that with a decreasing injected dose, the noise level in PET images increases, thus at lower doses, images become noisier [51]. This is also supported in Fig. 6, showing that the correlation between GQR and injected dose is stronger than for PR and DC. However, PR and DC, are more subjective metrics that involve the clinician using the images to make a diagnosis and judging their clinical plausibility based on the presence of specific pathological patterns, which is not provided as prior information to the network. In this case, the clinician estimated being able to make a reasonably confident diagnosis based on some of the noisier images. It has also been shown in Table III that it is not possible to only use injected dose standardised by patient weight as input into a machine learning network. Despite this model being much smaller, and using an easily available label, the best results were achieved when using PET images in the transfer learning dataset, evident by the number of green boxes in the table. Whilst most prior research is on binary classification and not in PET imaging, our maximum Spearman’s rank correlation coefficients of 0.96 for GQR, 0.67 for PR and 0.59 for DC are comparable to that achieved by Coyner *et al*.[43] (0.86 − 0.92).

Future work may include training and testing with a larger number of clinically-scored images, to observe the trend beyond seven separate transfer learning datasets. This work could also be applied to other low-dose reconstruction algorithms such as KEM [7] and MR-guided methods [52] or novel regularisation strategies [53]. As these methods are superior to standard OSEM, they should give higher quality predictions, even at lower simulated doses. However, similarly to [31], this may need to involve the inclusion of reconstructions generated via these algorithms into the training dataset. Further work could also investigate different established CNN backbones. Additionally, the model developed here may be used in different applications, including dynamic PET (where shorter frames are desirable for better time resolution, but also lead to noisier data) and different radioligands. Other recent advances, such as self-supervised learning for pre-training, as used by [54], [55], or vision-and-language pre-training for the similar task of determining clinical evaluations of medical images [56] could be investigated in relation to this clinical task.

## Conclusion

This study shows that pre-training can help improve network performance in the task of automatically predicting clinical image quality assessments of PET images. By transfer-learning using ImageNet weight initialisation and retraining the last two convolutional blocks of a VGG16 backbone, a superior model performance is achieved for this clinical task. Additionally, this method does not require further independent patient datasets, unlike for using a pretext learning task. Overall, this work shows that pre-training on natural images for application to medical images has the potential to provide benefit for regressing from images to clinically-scored metrics.

## Figures and Tables

**Table 1:** Correlation between clinical quality metrics

Metric Comparison	Pearson’s Correlation Coefficient	
	*Clinician 1*	*Clinician 2*
*GQR vs. PR*	*0.90*	*0.93*
*GQR vs. DC*	*0.84*	*0.93*
*PR vs. DC*	*0.96*	*0.99*

**Table II:** Pretext learning task: dose inference evaluation

VGG16 Network	Pre-training Weights	Mean Absolut Error	%Exact Agreement
Retrain last 2 blocks	ImageNet	0.09	71
Retrain all 5 blocks	Random	0.27	29

**Table III:** Comparison to easily accessible labels as input

Metric	Pre-trained Network			Dose/Weight to Clinical Scores (LR = 10^-3^)			Dose/Weight to Clinical Scores (LR = 10^-4^)		
	GQR	PR	DC	GQR	PR	DC	GQR	PR	DC
*% Exact Agreement*	56	22	11	0	0	0	0	0	0
*% Close Agreement*	89	44	33	89	56	33	78	56	33
*MAE*	0.20	0.64	0.71	0.29	0.65	0.89	0.28	0.64	0.88
*Spearman’s Rank*	0.97	0.64	0.52	0.39	0.66	0.64	0.39	0.66	0.64

**Table IV:** Comparison of clinical metrics for each pipeline

Model	Weight Initialisation	Trainable Layers	Mean Absolute Error ± Standard Deviation			Percentage Exact Agreement ± Standard Deviation			Percentage Close Agreement ± Standard Deviation			Spearman’ Rank Cirrelation Coefficient		
			GQR	PR	DC	GQR	PR	DC	GQR	PR	DC	GQR	PR	DC
VGG16 Backbone (No pre-training, LR = 0.001)	Random	All	0.72 ± 0.24	0.90 ± 0.17	0.99 ± 0.17	11 ± 11	3 ± 8	3 ± 8	52 ± 22	23 ± 8	16 ± 13	0.15	0.19	0.14
VGG16 Backbone (No pre-training, LR= 0.0001)	Random	All	0.51 ± 0.42	0.61 ± 0.07	0.63 ± 0.05	32 ± 18	30 ± 12	25 ± 11	75 ± 22	44 ± 6	51 ± 6	0.60	0.62	0.57
VGG16 Backbone (pre-training)	ImageNet	Last 2 Blocks and Fully Connected Layer	0.22 ± 0.06	0.66 ± 0.09	0.69 ± 0.05	52 ± 8	24 ± 8	27 ± 11	97 ± 5	49 ± 11	46 ± 10	0.96	0.67	0.59
Pretext Learning	Random Dose Inference	All	0.36 ± 0.01	0.64 ± 0.01	0.72 ± 0.01	11 ± 2	4 ± 10	13 ± 5	72 ± 10	64 ± 5	33 ± 0	0.93	0.54	0.59
Pretext Learning	ImageNet Dose Inference	Last 2 Blocks and Fully Connected Layer	0.32 ± 0.06	0.58 ± 0.09	0.68 ± 0.05	13 ± 4	31 ± 5	11 ± 0	71 ± 11	44 ± 0	44 ± 0	0.93	0.59	0.56
